# Cardiogoniometry Compared to Fractional Flow Reserve at Identifying Physiologically Significant Coronary Stenosis: The CARDIOFLOW Study

**DOI:** 10.1007/s13239-018-0354-1

**Published:** 2018-04-12

**Authors:** Oliver I. Brown, Andrew L. Clark, Raj Chelliah, Benjamin J. Davison, Adam N. Mather, Michael S. Cunnington, Joseph John, Albert Alahmar, Richard Oliver, Konstantinos Aznaouridis, Angela Hoye

**Affiliations:** 1 Department of Academic Cardiology, University of Hull, Castle Hill Hospital, Daisy Building, Castle Road, Cottingham, HU16 5JQ UK; 2 Department of Cardiology, Castle Hill Hospital, Castle Road, Cottingham, HU16 5JQ UK

**Keywords:** Cardiogoniometry, Fractional flow reserve, Coronary artery disease, Vectorcardiography, Diagnostic accuracy

## Abstract

Cardiogoniometry (CGM) is method of 3-dimensional electrocardiographic assessment which has been shown to identify patients with angiographically defined, stable coronary artery disease (CAD). However, angiographic evidence of CAD, does not always correlate to physiologically significant disease. The aim of our study was to assess the ability of CGM to detect physiologically significant coronary stenosis defined by fractional flow reserve (FFR). In a tertiary cardiology centre, elective patients with single vessel CAD were enrolled into a prospective double blinded observational study. A baseline CGM recording was performed at rest. A second CGM recording was performed during the FFR procedure, at the time of adenosine induced maximal hyperaemia. A significant CGM result was defined as an automatically calculated ischaemia score < 0 and a significant FFR ratio was defined as < 0.80. Measures of diagnostic performance (including sensitivity and specificity) were calculated for CGM at rest and during maximal hyperaemia. Forty-five patients were included (aged 61.1 ± 11.0; 60.0% male), of which eighteen (40%) were found to have significant CAD when assessed by FFR. At rest, CGM yielded a sensitivity of 33.3% and specificity of 63.0%. At maximal hyperaemia the sensitivity and specificity of CGM was 71.4 and 50.0% respectively. The diagnostic performance of CGM to detect physiologically significant stable CAD is poor at rest. Although, the diagnostic performance of CGM improves substantially during maximal hyperaemia, it does not reach sufficient levels of accuracy to be used routinely in clinical practice.

## Introduction

Non-invasive assessment is universally recommended in the investigation of patients with suspected stable coronary artery disease (CAD).^5^,^10^ However, there is still an unmet need for a quick, easy and cost-effective test which can exclude patients in a primary care or outpatient setting, who do not require further investigation.

**Figure 1:**
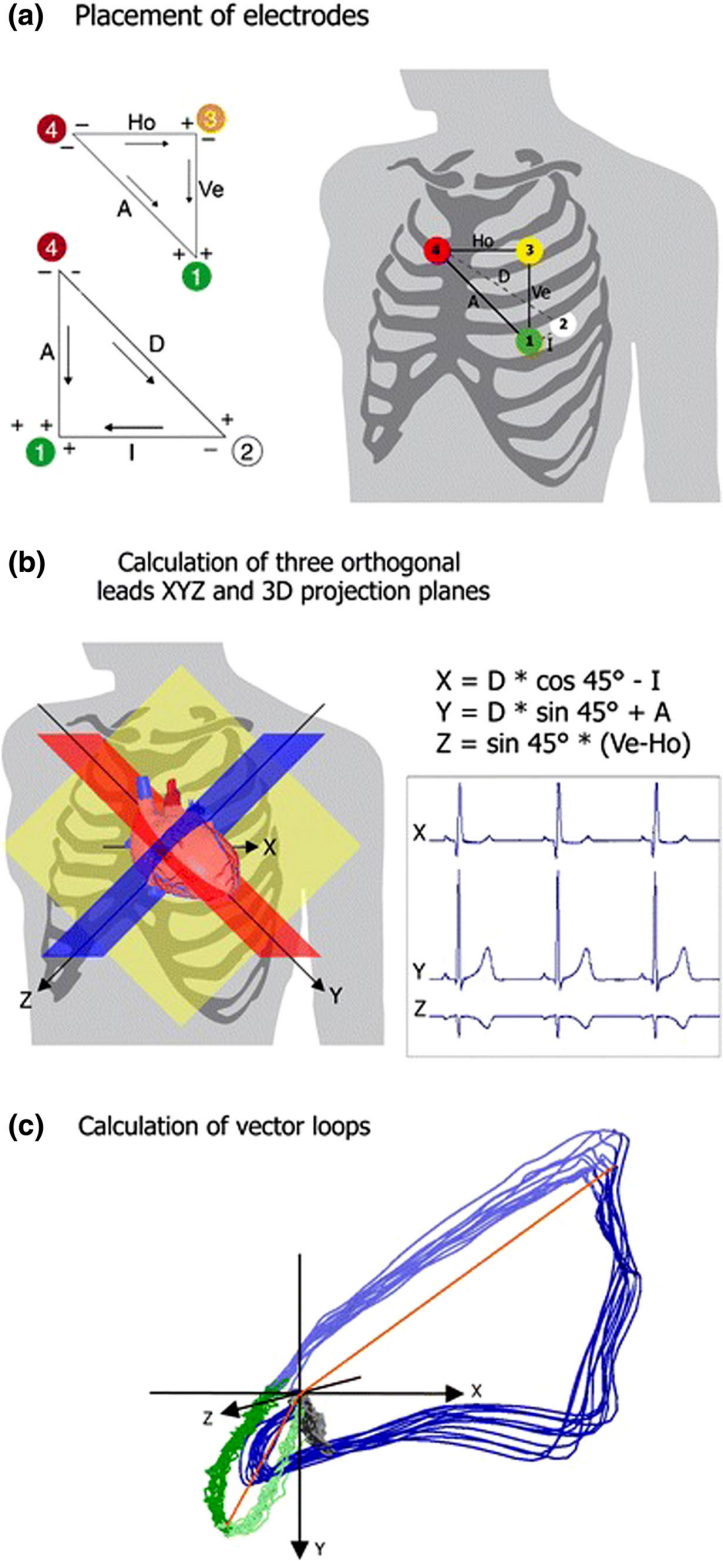
Principles of Cardiogoniometry: (a) Showing electrode placement: electrode 1 (green), Wilson position V4; electrode 2 (white), Wilson position V8; electrode 3 (yellow), directly superior to electrode 1 at a distance 0.7 times the distance between electrode 1 and 2; electrode 4 (red), directly right of electrode 3 at a distance the same as between electrode 1 and 3. The following leads are defined by the following electrodes: Anterior (A) by electrode 4 → 1; Horizontal (Ho) by electrode 4 → 3; Vertical (Ve) by electrode 3 → 1; Inferior by electrode 2 → 1 and Diagonal (D) by electrode 4 → 2 (see Fig. 1a). Triangles left of the thorax show the direction of the aforementioned leads. (b) Showing the orientation of orthogonal axes X, Y and Z in the thorax (left panel) and trigonometric equations defining their formation (right panel); (c) Showing the formation of vector loops by plotting of the heart vector at every millisecond for the P (grey), QRS (blue) and T waves (green), with maximum vectors for the P and QRS loop (orange lines) being shown. Reproduced from Tölg *et al*.^16^

Cardiogoniometry (CGM) is a method of 3-dimensional electrocardiographic assessment which uses five surface electrodes to produce a recording from three virtual bipolar leads (see Fig. 1). Importantly, an automated ischaemia scoring system that attributes a numerical value to the CGM recording, has been shown to have reasonable diagnostic performance at identifying angiographically defined, stable coronary artery disease (CAD).^7^,^13^,^20^ In addition, previous work has suggested the diagnostic performance of CGM is increased during adenosine stress testing.^19^ Nevertheless, angiographic evidence of CAD does not always correlate to physiologically significant CAD.^17^

Fractional flow reserve (FFR) is a technique used during cardiac catheterisation to assess the physiological significance of coronary artery stenoses. FFR works by comparing the ratio of blood flow distal to a coronary stenosis to normal flow during maximal hyperaemia.^11^ Previous work has shown that only 35% of coronary stenoses classed angiographically as 50–75% diameter stenosis are physiologically significant.^18^ Importantly, FFR guided percutaneous coronary intervention (PCI) has been shown to improve prognosis in patients with stable CAD.^17^

The aim of this prospective single centre, double blinded observational study was to evaluate whether non-invasive assessment with CGM could detect physiologically significant coronary stenosis defined by FFR, both at rest and during adenosine induced maximal hyperaemia.

## Methodology

### Study Participants

Forty-five patients with single vessel CAD admitted for elective PCI were consecutively recruited in a single tertiary centre between January and October 2016.

For inclusion, patients had to be aged 18 years or over and known to have single vessel CAD diagnosed on previous coronary angiography. All patients provided informed consent to undergo coronary angiography with pressure wire assessment ± PCI. Exclusion criteria were: patients with an acute coronary syndrome (as defined by the ESC^15^); patients unable to tolerate adenosine; patients unable to perform a good quality CGM; patients with atrial fibrillation; patients with haemodynamic instability and patients with previous coronary artery bypass graft surgery.

All subjects provided written informed consent to the study prior to enrolment. The study protocol was approved by the regional research ethics committee (12/YH/0271) and registered on www.clinicaltrials.gov, unique identifier: NCT02815631. The research project was conducted in accordance with the Declarations of Helsinki.

### Catheter Laboratory Protocol

A baseline CGM recording was performed in the catheter laboratory once the patient was relaxed and prior to commencing the invasive procedure. Radial or femoral access was then gained and patients were anticoagulated with 100 U/kg of heparin. A guide catheter was advanced to the coronary ostia as per usual clinical practice and coronary angiography performed of both the right and left coronary systems following administration of 200 mcg bolus of intra-coronary glyceryl trinitrate. The coronary pressure wire was advanced until the pressure sensor was aligned with the tip of the guide catheter, whereby both pressures were equalised. The coronary pressure wire was then advanced down the affected coronary artery being investigated and through the stenosis, where a bolus of 200 mcg of intracoronary glyceryl trinitrate was administered. Resting FFR and a second CGM recording were then performed. An intravenous adenosine infusion (180 mg/kg/min) was administered through a peripheral venous cannula in the antecubital fossa for 3 min or until maximal hyperaemia had been achieved. During maximal hyperaemia, peak FFR and CGM recordings were made. OIB then left the room to remain blinded to the result of the FFR assessment and took the CGM recordings for interpretation. The operating interventional cardiologist recorded the results of the FFR assessment and managed the patient as per clinical practice.

### Data Analysis

CGM data was recorded onto the Patient Explorer software version 2.1 [Enverdis, Jena, Germany]. The software automatically detects irregular or ectopic beats in the recording and excludes them from the analysis. All CGM recordings were interpreted by OIB, who remained blinded to the result of FFR. For each CGM tracing, the ischaemia score was recorded as a dichotomous result: negative (ischaemia score = 0) or positive (ischaemia score < 0).^13^

FFR was classified dichotomously as negative (FFR > 0.80) or positive (FFR ≤ 0.80) at baseline and at maximal hyperaemia by the operating interventional cardiologist.^17^

Both the CGM and FFR results were stored on separate encrypted electronic databases. At the end of participant recruitment, blinding was broken and OIB analysed the results of CGM (at rest and during maximal hyperaemia) in comparison to FFR (at maximal hyperaemia).

Quantitative coronary angiographic (QCA) analysis was performed using Centricity Explorer system. The reference vessel diameter, minimal lumen diameter (MLD), percent diameter stenosis and lesion length were measured before FFR assessment. Reference vessel diameter was taken as the diameter of the normal vessel proximal to the lesion.

It has been previously proposed that the diagnostic performance of CGM is driven by myocardial scarring, as opposed to chronic reversible ischaemia.^1^,^19^ Therefore, a pre-specified subgroup analysis for the diagnostic performance of CGM was patients without previous myocardial infarction. Finally, to allow comparison to previously published work, the diagnostic performance of CGM at rest was evaluated at identifying ≥ 50 and ≥ 70% diameter stenosis (DS).

### Statistical Analysis

IBM SPSS Statistics for Macintosh, Version 23.0 was used for statistical analysis. Descriptive statistics were used to summarise the data. Baseline continuous variables are expressed as mean ± SD or median with interquartile range, categorical data was expressed as numbers/percentages. Sensitivity, specificity, positive predictive value (PPV) and negative predictive value (NPV) were used to measure the diagnostic accuracy of CGM in comparison to FFR. Statistical agreement between CGM and FFR was calculated by the Kappa statistic.^3^

## Results

Baseline and angiographic characteristics for the study participants are shown in Tables 1 and 2 respectively. Eleven (24.4%) participants had previously had a myocardial infarction and 16 (35.6%) had previously received PCI. The majority of lesions were located in the left anterior descending artery (77.8%).

**Table 1:** Baseline characteristics of participants in CARDIOFLOW.

Demographics	
* N*	45
Male (%)	24 (53.3)
Age (SD)	61.4 (10.9)
Body mass index (SD)	30.5 (6.3)
Past medical history (%)	
Myocardial infarction	11 (24.4)
Percutaneous coronary intervention	16 (35.6)
Stroke/transient ischaemic attack	3 (6.7)
Diabetes mellitus	8 (17.8)
Hypertension	26 (57.8)
Hypercholesterolaemia	26 (57.8)
Smoking (never/ex/current)	16 (35.6)/19 (42.2)/10 (22.2)
Medication at enrollment (%)	
Aspirin	42 (93.3)
Clopidogrel	9 (20.0)
Ticagrelor	7 (15.6)
ACE inhibitor	13 (28.9)
Angiotensin receptor blocker	9 (20.0)
β-blocker	33 (73.3)
Ca^2+^ channel blocker	12 (26.7)
Lipid lowering drug	35 (77.8)
Baseline blood results	
Haemoglobin, g/L (SD)	141.4 (12.3)
Sodium, mmol/L (SD)	137.5 (2.3)
Potassium, mmol/L (SD)	4.3 (0.3)
Urea, mmol/L (SD)	5.9 (1.7)
Creatinine, mmol/L (SD)	78.8 (17.6)

Continuous data is expressed with its mean and standard deviation (SD)

**Table 2:** Showing the angiographic and fractional flow reserve (FFR) characteristics of study participants.

Angiographic details	
Stenosis site (%)	
LAD	35 (77.8)
RCA	4 (8.9)
LCX	3 (6.7)
OM	1 (2.2)
D	2 (4.4)
Stent implanted (% yes)	17 (37.8)
Reference vessel diameter (± SD)	2.96 ± 0.68 mm
Minimal luminal diameter (± SD)	1.27 ± 0.48 mm
% diameter stenosis (± SD)	56.90 ± 15.34
Lesion length (± SD)	18.49 ± 13.61 mm
FFR details	
Rest FFR (± SD)	0.90 ± 0.12
Peak hyperaemia FFR (± SD)	0.81 ± 0.13
Positive FFR at rest (%)	3 (6.7)
Positive FFR during hyperaemia (%)	18 (40.0)

Stenosis site is categorised into left anterior descending artery (LAD); right coronary artery (RCA); left circumflex artery (LCx); obtuse marginal artery (OM) and diagonal artery (D). Continuous data is expressed with its mean and standard deviation (SD)

At baseline, 16 (35.6%) participants had a positive CGM result which increased to 24 (53.3%) during maximal hyperaemia. Of the forty-five patients recruited in our study, eighteen (40%) were found to have significant coronary artery disease when assessed by FFR.

### Diagnostic Performance of CGM

The diagnostic performance of CGM to detect physiologically significant stenosis is shown in Table 3. At rest, the diagnostic performance of CGM was poor across all measures. During maximal hyperaemia the sensitivity of CGM significantly increased compared to rest (33.3 vs. 71.4%). However, a concomitant reduction in specificity was also observed (63.0 vs. 50.0%). PPV and NPV were also increased at maximal hyperaemia compared to at rest. No statistical agreement between CGM and FFR was found at rest (*κ* = − 0.04, *p* = 0.80). A fair agreement between CGM and FFR was seen during maximal hyperaemia, but this was not statistically significant (*κ* = 0.21, *p* = 0.14).

**Table 3:** Diagnostic performance of cardiogoniometry (CGM) to detect physiologically significant coronary stenosis.

	CGM at rest (*n* = 45)	CGM during maximal hyperaemia (*n* = 45)
Sensitivity	33.3%	71.4%
Specificity	63.0%	50.4%
Positive predicative value	37.5%	55.6%
Negative predicative value	58.6%	66.7%
Kappa statistic for agreement	− 0.04, *p* = 0.80	0.21, *p* = 0.14

The results of the subgroup analysis of patients without history of myocardial infarction is found in Appendix 1.

The diagnostic performance of CGM to detect CAD defined by % DS is shown in Table 4.

**Table 4:** Diagnostic performance of cardiogoniometry (CGM) to detect stable coronary artery disease defined as either ≥ 50% diameter stenosis (DS) or ≥ 70% DS.

Definition of stable CAD	CGM	
	≥ 50% DS	≥ 70% DS
Sensitivity	38.5%	16.7%
Specificity	70.0%	56.7%
Positive predicative value	76.9%	14.3%
Negative predicative value	30.4%	77.3%
Kappa statistic for agreement	0.06, *p* = 0.64	− 0.174, *p* = 0.22

## Discussion

The diagnostic performance of CGM to detect patients with physiologically significant stable CAD is poor at rest. Although, the diagnostic performance of CGM improves with the administration of intravenous adenosine, it does not reach sufficient levels of accuracy to safely exclude patients without physiologically significant stable CAD.

One of the main objectives of this study was to see if CGM had adequate sensitivity to safely stratify patients who required FFR assessment, avoiding the additional cost of the pressure wires and increased procedural risks. However, even during maximal hyperaemia the sensitivity of CGM was not high enough to be relied upon. For every hundred patients with physiologically significant stable CAD tested, CGM would not detect the presence of stable CAD in twenty-nine of those patients.

The reported figures for the measures of diagnostic performance of CGM in this study are considerably worse than previously published work which used coronary angiography to define CAD.^7^,^13^,^14^,^20^ In previously published CGM studies, CGM demonstrated greater sensitivity (64–72%) and specificity (60–82%) when ≥ 50% DS was used to define significant CAD then seen in our results. Furthermore, previous CGM studies which used ≥ 70% DS to define significant CAD, also reported a higher sensitivity (75–84%) and specificity (74–81%) than results seen in our study.^13^,^14^ The difference in diagnostic performance of CGM in our study compared to previously published work may be because the pre-test probability of having angiographic CAD was greater in our study, as the patients were known to have angiographic evidence of disease prior to being enrolled.

One major issue of the automated algorithm used by the CGM device is that when it was originally validated, it was based on data from a study where patients were classified as having stable CAD based only on coronary angiography (defined as ≥ 50% diameter stenosis).^13^ This is a fundamental flaw of the device algorithm, as % diameter stenosis is not a reliable method of detecting significant lesions causing ischaemia.^17^ This may explain the low specificity seen in our study, as the cardiogoniometric variables it classes as being present in patients with stable CAD are not sufficiently specific for physiologically significant coronary disease. Nevertheless, it should be stated that CGM was first developed before the routine use of FFR. Therefore, it is not unreasonable that its initial development was based on the ability of CGM to detect angiographically significant coronary stenoses, and not FFR significant stenosis.

All of the previously published work did not limit study participants to having single vessel disease and this may also contribute to explaining the observed differences in results. The diagnostic performance of CGM may be greater in patients with multi-vessel disease as they may have a greater ischaemic burden. Furthermore, patients with multi-vessel disease tend to have worse long term outcome, increased procedural risk and significant comorbidities.^4^ Nevertheless, this is the only study investigating CGM using FFR, a robust method of identifying physiologically significant myocardial ischaemia.

The diagnostic performance of CGM in this study was considerably worse than other methods of non-invasive physiological assessment of coronary ischaemia, including stress echocardiography and myocardial perfusion imaging (MPI).^6^,^12^ However, when these methods of physiological assessment have been assessed using FFR as the gold standard, they have been shown to have poor agreement with FFR to detect physiologically significant stable CAD.^8^,^9^ Reported figures in the literature of the sensitivity and specificity of stress echocardiography at identifying FFR-defined CAD are 50 and 90% respectively.^8^ MPI with SPECT has also been shown to perform poorly, with a sensitivity and specificity of 76 and 38% at identifying FFR-defined CAD.^9^ Of note, MPI with SPECT has been shown to overestimate FFR-defined ischaemia in 22% of participants and underestimate it in 36% of participants. An argument could be made that it is inappropriate to critique SPECT against FFR, as FFR was first validated against SPECT.^11^ However, since its validation, FFR based decision making has been shown to improve clinical endpoints and as such, can be taken as a reliable and robust method for identifying significant coronary stenoses.^17^ It is therefore difficult to criticise the diagnostic performance of CGM on the basis of other non-invasive methods of physiological ischaemia assessment.

It has previously been proposed that the diagnostic performance of CGM could be driven by detection of myocardial scarring, as opposed to chronic reversible ischaemia.^1^,^19^ It would be expected that the specificity of CGM would be reduced by myocardial scarring. However the results of this study have demonstrated that there were no significant differences in the specificity or sensitivity of CGM when patients with previous MI are excluded (Appendix 1). This correlates with previously published work, and we can therefore be confident that myocardial scarring does not have a big influence on the diagnostic performance of CGM.^19^

It would have been interesting to see if other stressing agents like dobutamine, may have increased the sensitivity of CGM. Dobutamine, a β_1_ adrenergic receptor agonist, acts by directly raising the metabolic demands of the myocardium by increasing heart rate and the force of cardiac contractility. Whereas the mechanism by which adenosine may induce ischaemia is less defined. It is likely indirect, and may be due to its ability to produce a coronary steal effect.^2^ As patients were already undergoing pharmacological stress with intravenous adenosine as part of their clinical care, it was felt to be unethical to subject them to an additional CGM stress test and induce the unpleasant side effects associated with dobutamine (nausea, headaches and dyspnoea). One previous study reports similar figures seen in this study for the sensitivity and specificity of CGM during adenosine stress.^19^ However, the authors of this study used SPECT to confirm the presence of stable CAD, which as previously mentioned is not a robust method of quantifying physiological significance. Interestingly, this previous study also showed a reduction in the specificity of CGM, when patients underwent pharmacological stress with intravenous adenosine.

CARDIOFLOW was designed to be as clinically applicable as possible, therefore the interpretation of the CGM result was based solely on the automated ischaemia score alone, and not by review of the raw data by an experienced CGM reporter. The idea being that, if CGM were to be implemented into routine clinical practice, a recording could be performed by an operator without detailed knowledge of CGM. Our patients are typical of routine clinical practice, a reflection of our consecutive recruitment of participants and reduction of the risk of selection bias. Furthermore, there was a wide range in both the length and severity of the lesions in the participants recruited in our study. This again mirrors the picture seen in routine clinical practice and increases the external validity of the study

### Study Limitations

This was a single centre study and only enrolled a relatively small number of participants. In addition to this, patients with multi-vessel disease, atrial fibrillation or previous CABG were excluded. Myocardial scarring was assumed in patients with previous MI and not formally assessed by performing cardiac MRI with late gadolinium enhancement; therefore, patients may have been incorrectly excluded from the subgroup analysis. Additional testing of participants with stress echocardiography and myocardial perfusion imaging would have allowed direct comparison between CGM and other methods of assessment, however this was not performed as it is not part of their routine clinical care. Finally, the operator performing CGM was not blind to the results of coronary angiography which could have influenced their interpretation. However, the result given by CGM is automated and dichotomous (i.e. categorically positive or negative) and therefore the impact of seeing coronary angiography would have been low.

## Conclusions

The diagnostic performance of CGM to detect physiologically significant stable CAD is poor at rest. Although, the diagnostic performance of CGM improves substantially during adenosine stress testing, it does not reach sufficient levels of accuracy to be used routinely in clinical practice.

## Appendix 1

See Table 5.

**Table 5:** Diagnostic performance of cardiogoniometry (CGM) to detect physiologically significant coronary stenoses when patients with previous myocardial infarction are excluded.

	CGM at rest (*n* = 34)	CGM during maximal hyperaemia (*n* = 34)
Sensitivity	41.7%	58.3%
Specificity	63.6%	54.5%
Positive predicative value	38.4%	41.2%
Negative predicative value	66.6%	70.6%
Kappa statistic for agreement	0.05, *p* = 0.76	0.12, *p* = 0.47
